# An Accommodation for Whom? Has the COVID-19 Pandemic Changed the Landscape of Flexible and Remote Work for Workers with Disabilities?

**DOI:** 10.1007/s10672-023-09472-3

**Published:** 2023

**Authors:** Jennifer D. Brooks, Sarah von Schrader

**Affiliations:** 1 Yang-Tan Institute on Employment and Disability, ILR School, Cornell University, 201 Dolgen Hall, Ithaca, NY 14853, USA

**Keywords:** Disability, COVID-19, Remote work, Flexible work, Race, Gender

## Abstract

At the start of the COVID-19 pandemic, flexible and remote work was viewed as a silver bullet that would increase employment rates among people with disabilities. This view fails to recognize that not all workers with disabilities can obtain jobs that can be done remotely or on a flexible schedule. Data from the 2019 and 2021 years of the Current Population Survey and the American Community Survey were used to examine if disabled workers’ gender, race, ethnicity, age, and education, increase (or decrease) their chances of accessing flexible and remote work and if the group of workers with disabilities who access such options expanded since the COVID-19 pandemic. Findings indicate that compared to their non-disabled counterparts, prior to the pandemic, workers with disabilities reported similar rates of flexible and remote work. Workers with disabilities, however, had lower rates of remote work after the start of the pandemic. Regardless of year, flexible and remote work rates vary by demographic group, with disabled workers who are white, female, and college-educated more likely to access these options than multiply marginalized disabled workers.

Roughly one-third of all working-age adults with disabilities in the United States were employed in 2022, compared with nearly three-fourths of those without disabilities (U.S. Bureau of Labor Statistics [BLS], 2023). This 40-percentage point employment gap between people with and without disabilities, which has persisted for decades, is a key indicator of the rampant marginalization that people with disabilities experience both within labor market contexts and society at large (Maroto & Pettinicchio, 2015). While some of this discrimination is the direct result of the stigma associated with having a disability, people with disabilities also experience various forms of state-sanctioned discrimination. For example, Sect. 14(c) of the Fair Labor Standards Act, a U.S. labor law passed in 1938 that is still on the books today, allows employers to pay employees with disabilities less than the minimum wage.

While laws such as the Americans with Disabilities Act (ADA) of 1990 make it unlawful to discriminate against individuals with disabilities, evidence suggests that disability-specific discrimination persists in the U.S. workplace. Over the last ten years, between 22,000 and 28,000 employment-related discrimination charges were filed annually with the U.S. Equal Employment Opportunity Commission (EEOC), the government entity charged with enforcing Title I of the ADA. These charges cite issues such as discriminatory discharge and failure to provide reasonable accommodations (US EEOC, n.d.).

There are multiple definitions of disability. Although disability has become more widely understood as socially constructed (created by a mismatch between one’s abilities and physical, social, and environmental barriers), misconceptions of people with disabilities persist. Research indicates that both non-disabled employers and coworkers perceive workers with disabilities as incapable, unwilling, or having poorer performance when it comes to work (Fraser et al., 2010). Simultaneously, many in the larger society view people with disabilities as dependent on government aid and a financial drain on the economy. Despite decades of disability activism that has proven these stereotypes false, these misconceptions still influence opportunities. While people with disabilities have demonstrated success in every industry, including business, politics, academia, and even filmmaking, these success stories are rare, masking the fact that most people with disabilities live in poverty and are chronically unemployed or underemployed (Maroto et al., 2019). These significant labor market disadvantages lead some scholars to assert that disability is an axis of inequality, similar to race, gender, and other status-based characteristics (Mauldin et al., 2020).

General U.S. employment rates, however, only tell half of this story. Recent evidence finds that the probability of employment varies widely among people with disabilities depending upon their race and gender, with the most marginalized people with disabilities experiencing the lowest employment probabilities (Brooks, 2021). Further evidence for this marginalization is seen in how the majority of the literature on the labor market inequalities of people with disabilities fails to address the issue of employment and disability from an intersectional perspective. That is, while there is a growing body of research on the labor market experiences of people with disabilities, only a handful of studies within this body of work considers the unique challenges faced by multiply marginalized people with disabilities, such as women and racially and ethnically diverse individuals.

One example of the consequences of this failure was seen in the early stages of the COVID-19 pandemic when some scholars argued that the rise in remote work options in the U.S would create new opportunities for people with disabilities, increasing their employment rates (Schur & Kruse, 2020). Proponents of this claim argued that the rise in the number of people working from home was likely to increase employers’ willingness to hire workers with disabilities because it would reduce employers’ need to accommodate these workers in the physical work environment (Schur & Kruse, 2020). Yet, this argument does not account for three essential facts about employment and disability: (1) people with disabilities face a web of policy, structural, and individual-level barriers when attempting to enter and maintain employment, (2) most workers with disabilities are segregated into low-wage/low-skilled jobs that cannot be done remotely (Maroto & Pettinicchio, 2014; Pettinicchio et al., 2022), and (3) While the EEOC has clarified that under the ADA flexible work options, like remote work and flexible schedules, can be a reasonable accommodation (US EEOC, 2003), not all people with disabilities have equal access to the reasonable accommodations they request (Harlan & Robert, 1998; Shuey & Jovic, 2013). These three fundamental principles may be exacerbated among multiply marginalized people with disabilities (Brooks, 2021). To address some of these points, we expand on prior work on the association between flexible /remote work and disability by examining how demographic characteristics, such as race, ethnicity, gender, age, and level of education, work in tandem with disability status to shape rates of these options.

Taking a more intersectional approach to the issue of flexible and remote work among people with disabilities leads to the question of which workers have access to (and take advantage of) such options. In other words, are flexible and remote work options only available to a select group of people with disabilities? To answer this overarching question, we examined how U.S. rates of flexible work hours and remote work are shaped by an individual’s disability status in combination with their other status-based characteristics. We used two nationally representative U.S based data sets to estimate percentages of participation in flexible and remote work, stratified by several individual-level characteristics, including disability status, gender, race/ethnicity, age, educational attainment, and combinations of these characteristics. We focused our analysis on three specific measures of flexible and remote work: flexible work hours, formal work from home arrangements, and fully remote work. Because both formal work-from-home arrangements and fully remote work indicate some form of remote work arrangement, we sometimes discuss these two measures together under the umbrella of remote work. Using these measures, we seek to address three primary questions:

1. Are workers with disabilities accessing flexible and remote work options more than their non-disabled counterparts?
2. Do status-based characteristics, such as an individual’s gender, race, age, and educational attainment, increase (or decrease) the chances that workers with disabilities will access flexible and remote work options?
3. Did the group of workers with disabilities who access such options expand during the COVID-19 pandemic?

## Literature Review

### Disability, Employment, and Remote Work

While disability has been defined in various ways from biological abnormalities to social construction, many social scientists have come to agree that disability is a byproduct of both biology and society. That is, while an individual’s mental and physical impairments are primarily rooted in biology, disability is shaped by the interaction between those impairments and the social world (Verbrugge & Jette, 1994). This definition points to the fact that people with physical and mental impairments are treated differently (discriminated against) in society.

This discrimination plays out within labor market contexts. In 2021, there was nearly a 40 percentage point employment gap between working-age adults ages 21 to 64 with and without disabilities in the US (41% vs. 79%) (Erickson et al., 2023). This employment gap has persisted in the US for decades and is the result of multiple policy, structural, and individual-level factors (Pettinicchio et al., 2022). For instance, while disability-specific government assistance programs, such as Supplemental Security Income (SSI) and Supplemental Security Disability Income (SSDI), provide critical medical and community living supports for people with disabilities, these programs have strict income and asset limits that force people to choose between stable employment and life-sustaining supports and services (that private insurers do not offer), such as wheelchairs, hospital visits, medications, substance abuse treatment, mental health counseling, and attendant care (Pettinicchio et al., 2022).

Those who can bypass the work disincentives of these government assistance programs face other structural barriers when attempting to navigate the labor market. For instance, many candidates with disabilities are excluded from the workforce even before landing an interview (Ameri et al., 2018). The select few that obtain gainful employment often face disability-related discrimination in the workplace, including bullying and harassment, exclusion, and denial of needed accommodations (Cook et al., 2019; Robert & Harlan, 2006). The built environment may pose another challenge for (potential) workers with disabilities through factors such as inaccessible workplaces and lack of accessible transportation (Crooks, 2007). Individual characteristics, such as race, gender, age, and educational attainment, exacerbate—or reduce—the effects of these policy and structural barriers (Maroto et al., 2019).

During the COVID-19 pandemic, some scholars argued that the rise in flexible and remote work options would shrink the employment gap between people with and without disabilities (Schur & Kruse, 2020). Indeed, studies on both flexible and remote work have pointed to their numerous benefits. Compared to traditional workers, flexible and remote workers report higher job satisfaction and retention rates, productivity gains, and better work-life balance (Hickox & Liao, 2020; Orr & Savage, 2021). These arrangements may be even more beneficial to specific groups of workers, including caregivers, those who work at a significant distance from the office, and people with disabilities (Kanter, 2022; Orr & Savage, 2021; Ozimek, 2020). Among workers with disabilities, flexible and remote work have been shown to improve job satisfaction and perceived job quality (Giovanis & Ozdamar, 2019). Workers with disabilities who have flexible or remote work arrangements also report fewer work absences, better work-life-balance, greater ability to work around impairment-related needs (i.e., doctors’ appointments, extended breaks, etc.), and have a reduced need for travel compared to those with disabilities who do not have such arrangements (Giovanis & Ozdamar, 2019; Igeltjørn & Habib, 2020; Kanter, 2022). Flexible and remote work may also reduce the number of ableist interactions, such as disability-based workplace bullying and harassment (Cook et al., 2019; Davis, 2021). Some research has suggested that remote work may increase employment opportunities for people with disabilities by allowing disabled workers, who otherwise may not be able to either travel to or access a physical work site, to work from home (Igeltjørn & Habib, 2020; Kanter, 2022).

Despite their various advantages, flexible and remote work have several potential disadvantages. Regardless of disability status, some flexible and remote workers report feeling socially isolated, lack of access to promotions, and difficulty navigating communication with colleagues and supervisors (Hickox & Liao, 2020; Orr & Savage, 2021; Schur et al., 2020). Studies have also pointed to disparities in remote work across race and level of education, where racial minorities and people with less education have less access to remote work opportunities than their more privileged counterparts (Orr & Savage, 2021; Schur et al., 2020). We expand upon this research by examining how these status-based characteristics come together to shape rates of flexible and remote work for people with disabilities.

Prior to the COVID pandemic in 2019, less than 6% of employed individuals in the US worked primarily from home (Coate, 2021). While research shows that a higher percentage of workers with disabilities worked remotely before the pandemic than those without disabilities (Schur et al., 2020), other studies indicate that many workers with disabilities were denied remote work accommodations. In fact, between 1995 and 2020, approximately two-thirds of courts denied disabled workers’ remote work accommodation requests (Hickox & Liao, 2020). Judges have cited several reasons for these denials, including lack of supervision and remote workers’ inability to work effectively in teams (Hickox & Liao, 2020; Kanter, 2022).

As COVID cases rose in the United States in 2020, the percentage of remote workers increased from 6 to 35%, or 48.7 million workers (Coate, 2021). Although in a post-pandemic society there may be a reversal of pre-pandemic decisions regarding remote work accommodations (Hickox & Liao, 2020; Kanter, 2022; Travis, 2021), some scholars have argued that these potential reversals may not have much of an impact on the working conditions of most employees with disabilities (Brooks, 2020). Research has pointed to the fact that many workers with disabilities are in jobs that cannot be done remotely (Kanter, 2022; Maroto & Pettinicchio, 2014; Schur et al., 2020), which may explain why there was a substantial remote work gap between workers with and without disabilities during the pandemic (Kruse et al., 2022). In May 2020, only 25.7% of U.S. employees with disabilities worked remotely, compared to 35.8% of those without disabilities (Kruse et al., 2022). Indeed, recent research on this topic has indicated that the rise in flexible and remote work among disabled workers occurred primarily in white-collar industries, such as management, business, financial, and administrative occupations, in which few people with disabilities are employed (Kruse et al., 2022; Schur et al., 2020).

Thus, we hypothesize that:

#### Hypothesis 1

Prior to the COVID-19 pandemic, U.S. workers with disabilities will have lower percentages of flexible and remote work than those without disabilities.

#### Hypothesis 2

During the COVID-19 pandemic, U.S. workers with disabilities will have lower percentages of flexible and remote work than those without disabilities.

### Disability, Remote Work, and Intersectionality

In addition to disability status, other demographic characteristics, such as gender, race/ethnicity, age, and education, also shape rates of flexible and remote work. In fact, some U.S.-based workers were more likely to switch to remote work during the height of the pandemic than others. Specifically, younger workers, women, Non-Hispanic whites, Asian Americans, those with a college degree, those with children, workers from the Northeast, and those in jobs that could be done remotely were disproportionately more likely to work from home during the pandemic (Coate, 2021; Kruse et al., 2022; Orr & Savage, 2021).

While all people inhabit multiple statuses at once, most studies addressing disability and labor market inequality construct a raceless, genderless, heteronormative disabled subject (Brooks, 2021). Recent work on the employment and economic inequalities of people with disabilities has questioned disability as a homogeneous experience paradigm, pointing to the fact that those most marginalized within the community, specifically women and racial minorities with disabilities, have worse economic and labor market outcomes than their more privileged disabled counterparts (Brooks, 2021; Brown & Moloney, 2019; Maroto et al., 2019). At the root of this new line of research is a commitment to intersectionality—the acknowledgment that individuals’ multiple statuses come together to shape their labor market experiences.

A term first popularized by Black feminist scholars, such as Kimberlé Crenshaw, Patricia Hill Collins, bell hooks, and others, to highlight Black women’s invisibility (Crenshaw, 1989), intersectionality views status-based characteristics (i.e., race/ethnicity, gender, disability status, sexuality, etc.) as puzzle pieces. While individual pieces contain some information, the pieces must be assembled into a picture to fully understand how an individual’s status-based characteristics work in tandem to shape their experiences of social institutions, such as the labor market (Brooks, 2021). In other words, systems of oppression, such as racism, sexism, and ableism, intertwine within the labor market to create more disadvantages for individuals who possess multiple marginalized statuses (Brooks, 2021; Crenshaw, 1989).

For example, in 2021, there was nearly a four-percentage point employment gap between women and men with disabilities ages 21 to 64 in the US, favoring men (39% vs. 43%) (Erickson et al., 2023). There are even wider employment gaps for people with disabilities from some racial and ethnically diverse backgrounds compared to their white counterparts. For instance, statistics show that only 34% of Non-Hispanic Blacks with disabilities ages 21 to 64 were employed in 2021, compared with 43% of similar Non-Hispanic whites—an 8% point gap (Erickson et al., 2023). While there are various reasons for these disparities, a partial explanation may lie in the fact that because some communities, specifically racial and ethnic minorities, have historically been marginalized within the disability rights movement (Bailey & Mobley, 2019), they may not be as aware of their rights under the ADA, compared to their white counterparts. As a result, they may not be as likely to have access to the knowledge that would help them more easily navigate the labor market while disabled, such as the ADA’s mandate for employers to provide reasonable accommodations.

This lack of an intersectional perspective also is seen within the literature on remote work and disability. While studies on the prevalence of remote work among disabled workers point to the fact that only certain employees with disabilities, specifically those who work in white-collar occupations, have access to such options (Kruse et al., 2022; Schur et al., 2020), little attention has been given to examining the demographic characteristics of this divide. Specifically, evidence points to the fact that this division may have its roots in how particular individual characteristics (i.e., disability status, gender, race, etc.) come together to shape disabled workers’ employment trajectories (Brooks, 2021).

For instance, research that pairs disability status with only one other status-based characteristic finds that six years after leaving high school, disabled women (compared to men with disabilities) and disabled Non-Hispanic whites (compared to disabled racial minorities with disabilities) have higher postsecondary completion rates (Sanford et al., 2011). Thus, because many “good” jobs that can be done remotely require at least some form of a postsecondary credential, we would expect that disabled flexible and remote workers will be disproportionately female and Non-Hispanic white.

Other studies provide further evidence of this potential simultaneous overrepresentation of Non-Hispanic white and females and underrepresentation of men and racial minorities among disabled flexible and remote workers. For instance, research on earnings inequalities among workers with disabilities has found that racial minorities with disabilities earn significantly less than their Non-Hispanic white counterparts (Maroto et al., 2019), suggesting that these individuals are less likely to be in the higher status jobs that provide employees access to flexible and remote work options. Further, 2016 BLS occupational data indicates that men with disabilities are more likely to work in production, transportation, and material moving occupations than women with disabilities (The Economic Daily, 2017), pointing to the fact that men with disabilities may be less likely to be in jobs that offer flexible and remote work arrangements than their female counterparts. Studies on the demographic characteristics of remote workers in the general population support these conjectures, finding racial, educational, and gendered disparities in remote work during the pandemic (Coate, 2021; Orr & Savage, 2021).

Thus, we hypothesize that:

#### Hypothesis 3

Certain disabled workers, specifically women, Non-Hispanic whites, younger workers, and those with higher levels of education, will report higher percentages of flexible and remote work than other disabled workers.

#### Hypothesis 4

The rise in remote work rates during the COVID-19 pandemic will result in a more diverse pool of workers with disabilities participating in flexible and remote work.

In sum, we suspect that flexible and remote work options are only available to a small select group of workers with disabilities. While the COVID-19 pandemic may have increased access to such options, we predict that the demographic characteristics of flexible and remote workers with disabilities will remain largely the same. We test these predictions by using two U.S.-based nationally representative data sets to compare 2019 and 2021 rates of flexible and remote work for workers with and without disabilities. These results were further stratified by certain demographic characteristics, including gender, race/ethnicity, age, and education.

## Methods

To capture how disability status interacts with other demographic characteristics to shape flexible and remote work rates in a U.S.-based context, we used data from two large nationally representative surveys: the Current Population Survey (CPS) Disability Supplement and the American Community Survey (ACS). For the CPS analysis, we combined data from 2019 to 2021, the most recent years that the CPS administered its disability supplement. We also analyzed data from the 2019 and 2021 1-year public-use files of the ACS to capture how rates of remote work may have shifted due to the COVID-19 pandemic.

The CPS Disability Supplement is designed to provide information to support policy-makers to improve employment outcomes for people with disabilities and is collected by the U.S. Bureau of Labor Statistics (BLS) as a supplement to the CPS Basic Monthly Survey. The Disability Supplement was fielded in 2012, 2019, and 2021. Because the CPS disability supplement was not conducted in 2020, we combined the two most recent waves from 2019 to 2021. We chose to combine these waves so we would have a large enough sample size to estimate flexible and remote work rates for smaller demographic groups.

The ACS is a cross-sectional nationally representative survey administered annually by the U.S. Census Bureau. It is one of the largest sources of disability data in the United States, allowing researchers to examine smaller sub-groups of people with disabilities. Because of its large numbers of persons with disabilities, robust employment measures, and its ability to capture those who work primarily from home, the ACS is well-suited for this analysis.

### Sample

To estimate employment rates by disability status in Table 1, we restricted the ACS samples to the civilian, non-institutionalized population ages 18 to 64. For our flexible and remote work analyses, we further limited the CPS and ACS samples to workers who were not self-employed (Tables 2, 3 and 4).

### Flexible and Remote Work

We centered our analysis around three measures of flexible and remote work. The CPS contains two of these measures. First, the CPS captures respondents with flexible work hours with the question, “Do you have flexible work hours that allow you to vary or make changes in the time you begin and end work?” Those with formal work from home arrangements were identified with the question, “Do you have a formal arrangement with your employer to be paid for the work that you do at home, or were you just taking work home from the job?” The ACS employs a different method for capturing those who work from home, identifying respondents as fully remote workers if they state that they, “work at home” when asked about their means of transportation to work.

### Disability and Demographic Characteristics

The CPS and the ACS use the same six-item disability question sequence to identify respondents with physical and mental limitations (Brault, 2009). This sequence contains items for serious difficulties with, “hearing,” “seeing even when wearing glasses,” “walking or climbing stairs,” “concentrating, remembering, or making decisions,” “dressing or bathing,” and “doing errands alone such as visiting a doctor’s office or shopping.” Those who responded in the affirmative to any of these six items were coded as having a disability.

We incorporated several demographic characteristics in our analysis. Gender is a binary variable, with one as female and zero as male. While we recognize that there are many other genders, and gender itself does not exist as a binary, the ACS only includes the categories of male and female. Race/ethnicity includes four categories, Non-Hispanic white, Non-Hispanic Black, Non-Hispanic other, and Hispanic. Although there were several ways that we could have constructed these race/ethnicity categories, we chose to construct these four variables to ensure we had sufficient numbers for our analysis. Age was divided into three categories: 18–39, 40–50, and 51–64. We also constructed four levels of educational attainment: less than a high school education, high school degree (or GED), some college, and four-year college degree or beyond.

### Analysis

We began our analysis by examining employment rates for both the 2019 and the 2021 ACS samples by disability status. We also examined how employment rates for people with and without disabilities varied by gender, race, ethnicity, age, and educational attainment (Table 1). Next, we used data from the CPS to estimate percentages of employees with flexible work hours and work from home arrangements stratified by disability status. These percentages were further stratified by other key demographic factors (Table 2). Finally, for Tables 3 and 4, we estimated percentages of fully remote work for all possible 2- and 3- way interactions between the demographic characteristics, stratified by disability status. These estimates allowed us the flexibility to address each of our hypotheses, however, logistic regression analyses for the demographics regressed on each flexible work outcomes were also conducted and are available from the authors upon request. All estimates were calculated using the appropriate weights to represent the target population, and 95% confidence intervals (CI) were calculated using replicate weights as described in the ACS and CPS technical documentation. Analyses were conducted within STATA 17.1 and SAS 9.4.

**Fig. 1:**
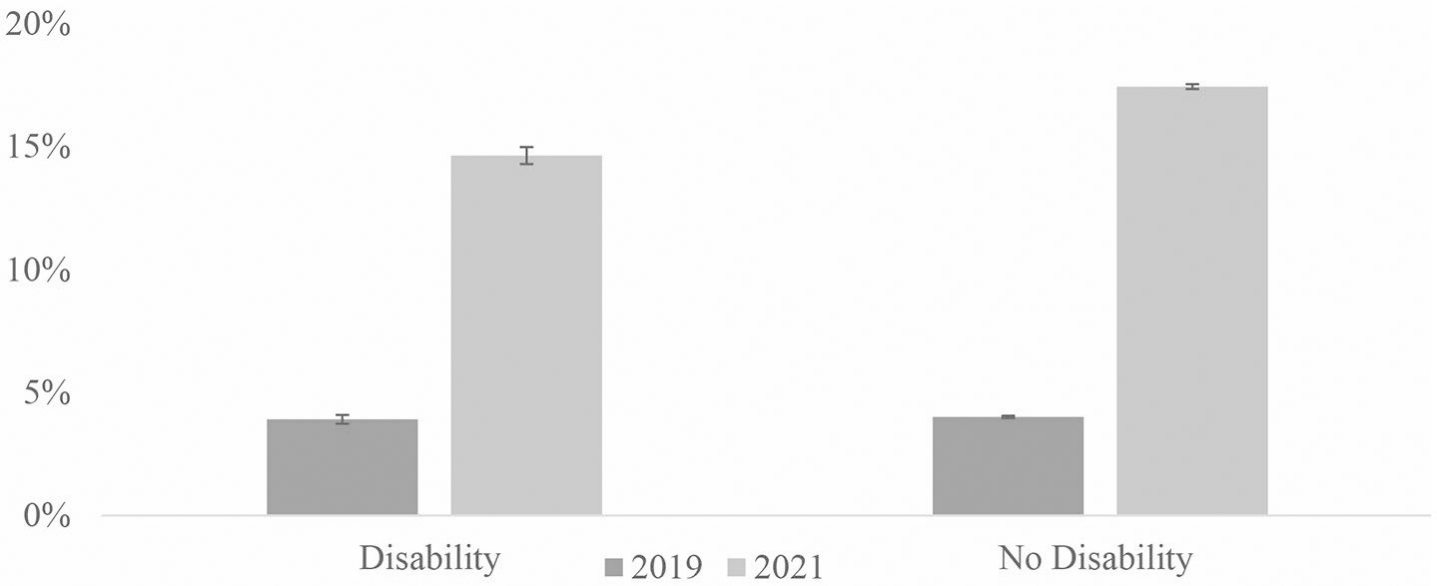
Percentage of workers reporting fully remote work by year and disability status. Sources: American Community Survey (ACS) 2019 and 2021 NOTES: Includes the civilian non-institutional population of workers who were not self-employed, ages 18 to 64. Includes 95% CI bars

**Fig. 2:**
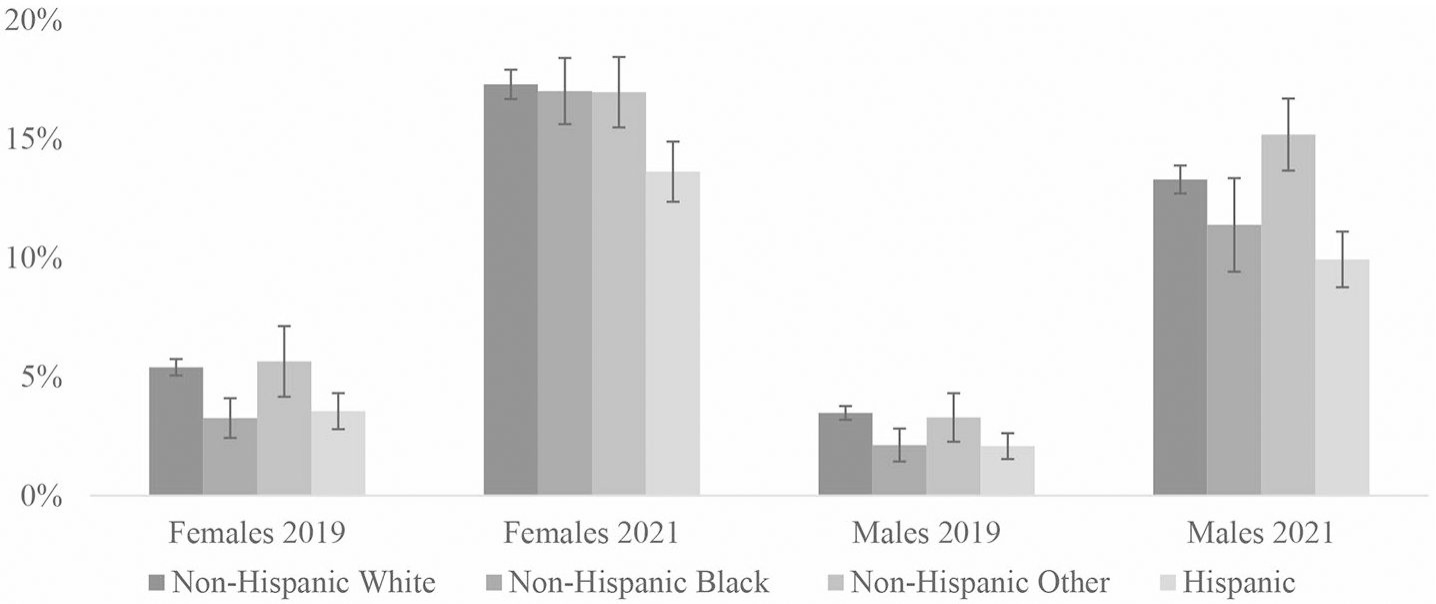
Percentage of workers with disabilities reporting fully remote work by gender, race/ethnicity, and year. Sources: American Community Survey (ACS) 2019 and 2021 NOTES: Includes the civilian non-institutional population of workers who were not self-employed, ages 18 to 64. Includes 95% CI bars

**Fig. 3:**
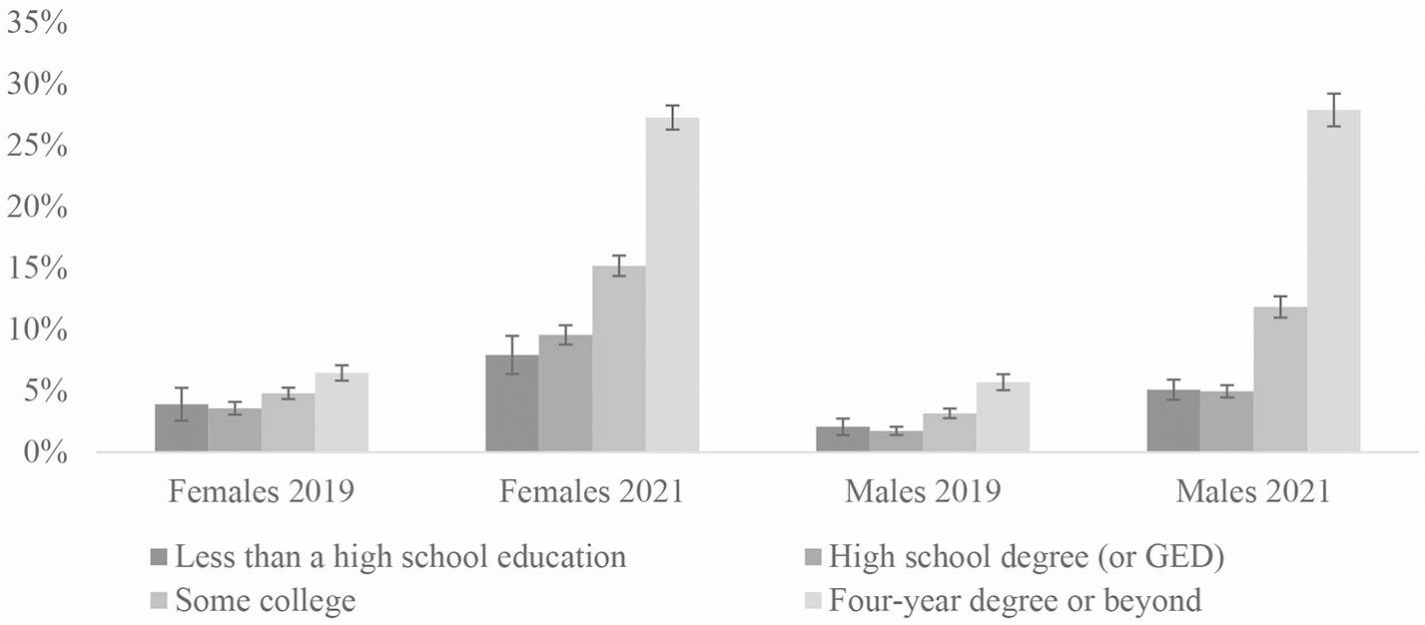
Percentage of workers with disabilities reporting fully remote work by gender, education, and year. Sources: American Community Survey (ACS) 2019 and 2021 NOTES: Includes the civilian non-institutional population of workers who were not self-employed, ages 18 to 64. Includes 95% CI bars

**Fig. 4:**
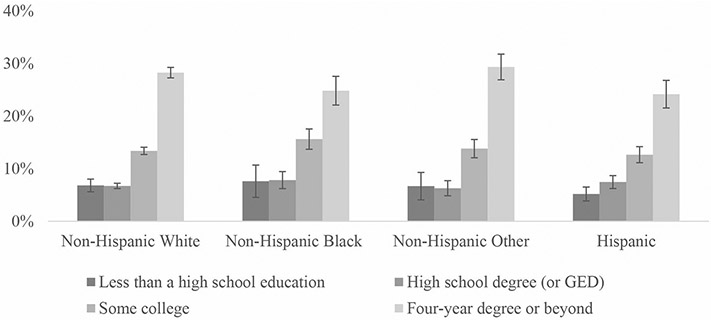
Percentage of Workers with Disabilities Reporting Fully Remote Work by Race-Ethnicity and Education (2021). Sources: American Community Survey (ACS) 2021 NOTES: Includes the civilian non-institutional population of workers who were not self-employed, ages 18 to 64. Includes 95% CI bars

To supplement our tables, we included several figures showing fully remote work rates by the various demographic groups. Figure 1 compares fully remote work rates by year and disability status. Figure 2 shows rates by gender, race/ethnicity, and year. Figure 3 shows how rates differ for women and men with disabilities by education group. Finally, Fig. 4 shows 2021 fully remote work rates by race /ethnicity and education. All figures include 95% CI bars.

## Results

Table 1 presents the percentage of adults ages 18 to 64 who were employed (including those who were self-employed) in 2019 and 2021 by disability status and other key demographic characteristics. According to this table, in 2019, 38.8% (95% CI [38.6, 39.1]) of individuals with disabilities were employed, compared to 78.6% (95% CI [78.5, 78.7]) of those without disabilities. Regardless of disability status, women were less likely to be employed than men, Non-Hispanic Black Americans were less likely to be employed than Non-Hispanic whites, and those with lower levels of education were less likely to be employed than those with a four-year college degree or beyond.

Compared to 2019 rates, 2021 employment rates were higher for people with disabilities (38.8% (95% CI [38.6, 39.1]) vs. 40.7% (95% CI [40.4, 41.0])) but lower for those without disabilities (78.6% (95% CI [78.5, 78.7]) vs. 76.6% (95% CI [76.5, 76.7])). In 2019 and 2021, women had lower employment rates than men, and Non-Hispanic Blacks had lower rates than Non-Hispanic whites, irrespective of disability status. Among people with disabilities, both 2019 and 2021 employment rates increased with education but decreased with age.

As we address each of our research questions below, we discuss our three types of flexibility in the workplace: flexible work hours, formal work from home arrangements, and fully remote work arrangements.

### Question 1: Were workers with disabilities more likely to access flexible hours and remote work options than their non-disabled counterparts prior to COVID-19?

Prior to the COVID-19 pandemic, disabled workers reported similar (not significantly different) rates of flexible work hours, formal work from home arrangements, and fully remote work as those without disabilities, which is contrary to our expectations. (Hypothesis 1). As shown in Table 2, in 2019, about 37.9% (95% CI [33.3, 42.6]) of workers with disabilities had flexible work hours, compared to 34.3% (95% CI [33.2, 35.4]) of those without disabilities. Overall, rates of formal work from home arrangements were similar among workers with disabilities than those without disabilities (e.g., 11.2% (95% CI [(8.1, 14.3]) vs. 11.0% (95% CI [10.4, 11.5])).

While only a handful of employees worked fully remote in 2019, workers with and without disabilities reported similar rates of fully remote work (3.9% (95% CI [3.7, 4.1]) vs. 4.0% (95% CI [4.0, 4.1]), see Table 3; Fig. 1).

### Question 2: Do rates of flexible hours and remote work among people with disabilities vary by individual characteristics?

As predicted, among workers with disabilities, rates of flexible work hours and remote work arrangements varied by individual characteristics (Hypothesis 3 and 4). For instance, looking at data combined for 2019 and 2021, disabled female workers and those with disabilities with four-year college degrees or beyond had higher percentages of flexible hours and formal work from home arrangements than other disabled workers within their demographic categories. While 49.0% (95% CI [42.3, 55.6]) of disabled workers with at least a four-year college degree had flexible work hours, this percentage declined to only 29.3% (95% CI [17.6, 41.0]) for those without a high school diploma. Aligned with our expectations, 19.6% (95% CI [15.7, 23.6]) of women with disabilities had formal work from home arrangements, compared to only 13.4% (95% CI [10.2, 16.6]) of men with disabilities.

Further evidence for this demographic divide between disabled workers who access remote work options and those who do not was found when examining how various intersecting statuses come together to shape disabled workers’ rates of fully remote work arrangements in Tables 3 and 4. As expected, in 2019, certain demographic sub-groups had higher percentages of fully remote work arrangements than others (Table 3; Hypothesis 3). For instance, among the various gender-race intersections, women with disabilities who were either Non-Hispanic white (5.4% (95% CI [5.1, 5.7])) or identified as other races (5.6% (95% CI [4.5,7.1])) had higher percentages of fully remote work arrangements than Non-Hispanic Black (3.3% (95% CI [2.6, 4.1])) and Hispanic women (3.6% (95% CI [2.9, 4.3])) (Table 4; Fig. 2). Regardless of race and ethnicity, disabled men had lower percentages of fully remote work arrangements than their female counterparts (Table 4).

An individual’s gender and educational attainment also shaped their rates of fully remote work arrangements. For instance, regardless of age, disabled women had higher percentages of fully remote work arrangements than men with disabilities. The effects of gender on one’s probability of fully remote work arrangements can be seen in the various gender-education intersections as well. Women had higher percentages of fully remote work arrangements than their male counterparts, regardless of educational attainment (Fig. 3). Among the age-education groups, workers with disabilities with educational credentials beyond a high school degree or GED had higher rates of remote work than workers with a high school degree or less, regardless of age group.

### Question 3: Did the group of workers with disabilities who access flexible hours and remote work expand during the COVID-19 pandemic?

From 2019 to 2021, there was no noticeable expansion in the percentage of workers with disabilities with flexible work hours (Table 2). Contrary to our expectations, during the COVID-19 pandemic, the remote work rate increased more for workers without disabilities than for those with disabilities (Hypothesis 2). While workers with disabilities had similar formal work from home rates in 2019, by 2021, the rate for non-disabled workers was 2.7% points higher than for those with disabilities, although the difference was not statistically significant (23.7% (95% CI [22.7, 24.7]) vs. 21.0% (95% CI [(17.3, 24.8])) (Table 2). The fully remote work gap between employees with and without disabilities rose from 0.1 percentage points in 2019 to nearly 3 percentage points in 2021, with people without disabilities accessing remote work at higher rates. Although there was a substantial increase in fully remote work arrangements among all workers, percentages increased more for workers without disabilities (from 4.0% (95% CI [4.0, 4.1]) to 17.4% (95% CI [17.3, 17.5]) compared to workers with disabilities (from 3.9% (95% CI [3.7, 4.1]) to 14.6% (95% CI [14.3, 15.0])) (Table 3; Fig. 1).

Similar to pre-pandemic levels, 2021 fully remote work rates among those with disabilities varied by demographic characteristics. While there was an increase in the overall number of disabled workers who participated in remote work in 2021, this increase did not expand along demographic lines. That is, contrary to our expectations for Hypothesis 4, the rise in flexible and remote work among workers with disabilities did not create a more diverse pool of remote workers.

As shown in Table 4; Fig. 2, in 2021, disabled women who were Non-Hispanic white, Black, and other all still had higher fully remote work percentages than men from any race. Among workers with disabilities, Non-Hispanic white women (17.3% (95% CI [16.7, 17.9])) had the highest rates of fully remote work, while Hispanic men (9.9% (95% CI [8.9, 11.1])) had the lowest. Disabled women in any age group had higher percentages of fully remote work than men at any age. Among most of the gender-education groups, women with disabilities had higher rates of fully remote work than men with disabilities, except for those with a four-year college degree or beyond, where disabled men had slightly higher, but not significantly different rates of remote work than similarly educated disabled women (27.9% (95% CI [26.6, 29.2]) vs. 27.2% (95% CI [26.3, 28.2])) (Fig. 3).

Similar to results from 2019, disabled workers with higher levels of education had higher percentages of fully remote work. For instance, regardless of race, those with a four-year college degree or beyond had the highest rates of fully remote work within their race groups, while those with less than a high school education or a high school diploma/GED had the lowest. The effects of education on percentages of fully remote work were further emphasized when examining the various age-race-ethnicity groups where disabled Non-Hispanic Other workers with a four-year college degree or beyond had the highest percentages of fully remote work than any other three-way status combination (29.4% (95% CI [27.0, 31.8])) (Fig. 4).

## Discussion

The COVID-19 pandemic dramatically transformed how we think about work, both in the United States and internationally (International Labor Organization (ILO), 2023). While some workers benefited from this transformation, taking advantage of the normalization of flexible and remote work, other less privileged workers experienced a worsening of their working conditions, including increased hours, a high risk of COVID-19 exposure, and the increasing threat of unemployment (Paine, 2020). While some employment and disability scholars like to imagine that most workers with disabilities fall into the first category, our findings indicate otherwise. That is, our findings expand on prior work on the association between flexible /remote work and disability (Kruse et al., 2022; Schur et al., 2020) by revealing how certain groups of disabled workers, those who were female, Non-Hispanic white, or college-educated, had higher rates of participation in flexible and remote work during the pandemic when compared with other disabled workers, suggesting that the normalization of remote work (Travis, 2021) only benefited a select group of workers with disabilities. While our findings primarily apply to the U.S, we suspect that these patterns may also be found in other countries as well, given that most industrialized nations saw a similar rise in flexible and remote work during the COVID-19 pandemic (ILO, 2022).

Our study used data from the CPS and the ACS to examine rates of flexible work hours and remote work before and during the COVID-19 pandemic in the U.S and how these rates vary by certain status-based characteristics, such as race/ethnicity, gender, age, and educational attainment. Our results show that compared to their non-disabled counterparts, prior to the pandemic, workers with disabilities reported higher rates of flexible work hours and similar rates of remote work. Workers with disabilities, however, had lower rates of remote work after the start of the pandemic. We also found that disabled workers’ rates of flexible work hours and remote work vary substantially by demographic characteristics, with women, Non-Hispanic whites, and those with four-year college degrees or beyond reporting higher rates of flexible work hours and remote work than other disabled workers within their demographic categories. With few exceptions, these disparities still existed in 2021. Overall, our findings point to four main conclusions:

First, our results indicate that while workers with disabilities reported higher rates of flexible work hours, they were similarly likely to work remotely than their non-disabled counterparts prior to the pandemic. During the pandemic, however, workers with disabilities had lower rates of remote work than those without disabilities. These findings contradict prior studies that suggest that workers with disabilities are more likely to report remote work arrangements than those without disabilities (Schur et al., 2020).

Despite these divergent results, U.S-based literature on the labor market inequalities of people with disabilities provides substantial evidence in favor of our conclusions. For instance, research suggests that employers may perceive (and process) remote work requests differently depending on employees’ disability status. That is, from the perspective of employers, workers without disabilities may request remote work due to extenuating circumstances, such as increased childcare responsibilities, a temporary illness/injury, or other personal-related matters, and thus be more amiable to these requests. On the other hand, employers may view disabled employees’ remote work request (an accommodation) as a “special right” and may be less likely to grant such a request (Harlan & Robert, 1998; Hickox & Liao, 2020; Kanter, 2022). Indeed, research suggests that, in general, people without disabilities are more likely to view disability-specific accommodations as special rights or privileges rather than modifications needed to ensure that individuals with disabilities can fully participate in society (Dorfman, 2019).

Another factor that may explain why workers with disabilities report lower rates of remote work than their counterparts without disabilities is their greater vulnerability to occupational segregation (Maroto & Pettinicchio, 2014). That is, because workers with disabilities are more likely to be segregated into low-wage, low-skilled, precarious, non-unionized jobs than those without disabilities (Pettinicchio et al., 2022), they are less likely to be in jobs that can be done remotely. As a result, workers with disabilities may report lower rates of remote work simply because they are less likely to have access to these options.

Second, looking across years, we found that certain disabled workers had a higher prevalence of flexible work hours and remote work arrangements than others. Workers with disabilities who are female, Non-Hispanic white, Non-Hispanic other, or have a four-year college degree or beyond reported higher rates of flexible work hours and remote work compared to other disabled workers. This uneven distribution of flexible and remote work could be directly linked to the racial and gendered disparities in occupational type among workers with disabilities. Evidence suggests that even when employed in jobs that can offer flexible work hours and remote work options, workers with disabilities who identify as Non-Hispanic white are more likely to have their accommodation requests approved than similar disabled workers from racial minorities (Harlan & Robert, 1998).

Another factor that may explain the lower rates of flexible and remote work among racial and ethnic minorities with disabilities may be linked to how these communities understand disability and how these understandings inform access to information on disability-specific laws, like the ADA. Societies that have been built upon the foundations of capitalism and white supremacy, such as the United States, have linked the value of racial and ethnically diverse individuals to their ability to perform free and/or cheap labor. Because of this, disability within these communities is often constructed as a sign of weakness (Lockhart, 2019). This link between disability and weakness means that many of these individuals, who may be chronically ill or disabled, are reluctant to identify as such (Bailey & Mobley, 2019). This is compounded by the fact that racial and ethnic minorities with disabilities have traditionally been excluded from both the disability rights movement and outreach efforts among some disability rights organizations (Erkulwater, 2018). The reluctance to identify as disabled, along with their exclusion from disability rights conversations and spaces, limits many racial and ethnic minorities with disabilities’ access to (and knowledge of) disability-specific laws and policies. This lack of access may contribute to their lower rates of flexible and remote work because they may not be aware that the ADA allows for such options (Inclusively, 2022).

Third, when analyzing 2021 remote work data, we found that while rates of remote work increased for all workers during the pandemic, regardless of disability status, these rates increased more for workers without disabilities. Our pandemic analysis reveals that the disability remote work gap increased from 0.1% points in 2019 to 2.8% points in 2021. This increase could be explained by the fact that, in general, workers with disabilities are more likely to be segregated into low-wage/low-skilled jobs than those without disabilities (Maroto & Pettinicchio, 2014). Thus, as remote work rates increased among white-collar workers, these increases disproportionately benefited workers without disabilities.

Fourth and finally, despite the increase in rates of remote work among all workers, the group of disabled workers who reported the highest rates of remote work in 2021 remained predominantly female, white, and highly educated. While this continued overrepresentation of privileged disabled workers is likely the result of the link between job type and access to such arrangements, it also indicates another—slightly hidden—story about marginalized workers with disabilities. That is, while many privileged disabled workers seem to have taken advantage of remote work options during the pandemic, many other workers with disabilities, specifically men, racial minorities, and those with lower levels of education, may not have been able to take advantage of such options, due to being in more physically demanding, direct service work that requires they be on site.

This lack of access to remote work options among multiply marginalized workers with disabilities may have further increased their vulnerability to certain adverse economic and health-related outcomes during the pandemic (Maroto et al., 2019). Those who could maintain employment during the pandemic were also likely to have been at an increased risk of being exposed to COVID-19, which is more severe and leads to higher hospitalization rates and death among people with disabilities (Sabatello et al., 2020). Research also found that during the pandemic, workers with disabilities in precarious employment experienced increased rates of depression compared with their counterparts without disabilities (Brown & Ciciurkaite, 2022). These adverse economic and health outcomes experienced by multiple marginalized workers with disabilities during the pandemic will likely exacerbate inequalities among disabled workers and may reduce the population of people with disabilities who can even participate in paid employment.

### Limitations

Despite its contributions, our study is not without limitations. One of the most significant limitations relates to how the CPS and the ACS construct their disability measures. While these questions capture some people with disabilities, they do not capture the entire disabled population, including people with substance use disorders, chronic illnesses, those who identify as neurodivergent, people with developmental disabilities, and those who develop their disabilities from poor health, injuries, childhood trauma, or obesity (see Brooks, 2021). This six-question sequence also excludes people with moderate or mild disabilities (Landes et al., 2020). Further, the ACS/CPS disability questions cannot capture disability onset. Thus, while disability type, severity, and onset are all essential factors to account for when examining the labor market inequalities of people with disabilities, we could not include these factors in our analysis.

Finally, while we could account for some key individual characteristics, we were limited in the demographic variables we could include. For instance, neither the CPS nor the ACS asks respondents about their sexuality, gender identity, religion, or other lesser studied markers of identity. Thus, we could not include these factors in our analysis. Further, although the ACS is one of the largest sources of disability data, we were still limited in how we constructed our racial categories. Smaller racial categories, such as Native American Alaskan Native and Asians, became relatively small when introducing them into the three-way interactions. As a result, we combined these two racial categories with the category of Non-Hispanic other.

## Conclusion

Despite its limitations, through consideration of intersections of disability with other characteristics, our findings reveal how certain U.S. workers with disabilities use their power and privilege to access flexible and remote work accommodations while their less privileged counterparts cannot. While workers with disabilities make up roughly 6–7% of the U.S. labor force (United States Census Bureau, 2021), this statistic masks the diversity of experiences that they face in the workplace. Our results point to the fact that disability employment policy that is constructed as a one-size-fits-all model is insufficient for understanding the labor market experiences of disabled workers with other marginalized identities. Our findings suggest that workers’ ability to shape these experiences are linked with their gender, race, age, and education. Even when employed, the intersection of disabled workers’ other status-based characteristics shapes their experiences of the labor market, which is in line with prior research on how various demographic factors impact disabled workers’ employment experiences (Brooks, 2021; Brown & Moloney, 2019). As a result, it is imperative that employment policies and programs that target people with disabilities take the intersection of ableism, racism, sexism, classism, ageism, and other forms of oppression into account to create a labor market that is accessible for all.

## Figures and Tables

**Table 1:** Employment Rates by Disability Status and Other Characteristics (American Community Survey, 2019 and 2021)

	2019 Employment Rates (%)				2021 Employment Rates (%)			
	With Dis.	95% CI	No Dis.	95% CI	With Dis.	95% CI	No Dis.	95% CI
Disability	38.8	(38.6, 39.1)	78.6	(78.5, 78.7)	40.7	(40.4, 41.0)	76.6	(76.5, 76.7)
Gender								
Female	36.6	(36.3, 37.0)	73.8	(73.7, 73.9)	39.3	(38.9, 39.7)	71.9	(71.8, 72.0)
Male	41.1	(40.7, 41.4)	83.6	(83.5, 83.7)	42.2	(41.7, 42.6)	81.4	(81.3, 81.5)
Race/Ethnicity								
Non-Hispanic White	40.0	(39.7, 40.4)	80.2	(80.1, 80.3)	41.5	(41.2, 41.8)	78.7	(78.6, 78.8)
Non-Hispanic Black	32.1	(31.3, 32.9)	75.5	(75.2, 75.8)	33.8	(33.1, 34.4)	72.5	(72.1, 72.8)
Non-Hispanic Other	39.5	(38.5, 40.6)	75.7	(75.5, 76.0)	41.8	(41.0, 42.6)	74.4	(74.2, 74.6)
Hispanic	40.9	(40.2, 41.5)	77.1	(76.9, 77.3)	43.7	(43.0, 44.5)	74.3	(74.1, 74.5)
Ages								
18 to 39	46.2	(45.8, 46.7)	77.6	(77.4, 77.7)	47.9	(47.3, 48.4)	75.2	(75.1, 75.4)
40 to 50	42.3	(41.7, 43.0)	85.0	(84.9, 85.1)	44.6	(44.0, 45.3)	83.0	(82.9, 83.2)
51 to 64	32.4	(32.0, 32.8)	75.2	(75.1, 75.4)	33.7	(33.2, 34.1)	73.7	(73.5, 73.8)
Educational Attainment								
Less than a high school	23.8	(23.2, 24.3)	63.9	(63.7, 64.2)	23.9	(23.4, 24.5)	61.8	(61.5, 62.1)
High school diploma/GED	33.5	(33.1, 34.0)	75.0	(74.8, 75.2)	34.5	(34.0, 34.9)	71.8	(71.6, 72.0)
Some college	44.0	(43.5, 44.5)	77.8	(77.6, 77.9)	45.4	(44.9, 45.9)	75.0	(74.9, 75.2)
Four-year degree or beyond	58.8	(58.2, 59.5)	86.4	(86.2, 86.5)	61.5	(60.9, 62.1)	85.2	(85.1, 85.3)

Sources: American Community Survey (ACS) 2019 and 2021

NOTES: Includes the civilian non-institutional population, ages 18 to 64

**Table 2:** Percentage of Workers with a Formal Work from Home (WFH) Arrangement or Flexible Work Hours by Disability Status and Other Characteristics (CPS Disability Supplement, 2019 and 2021 combined)

	Formal WFH Arrangement				Flexible Work Hours			
	With Dis.	95% CI	No Dis.	95% CI	With Dis.	95% CI	No Dis.	95% CI
Overall	16.4	(13.9, 18.8)	17.2	(16.6, 17.8)	38.4*	(34.9, 41.8)	34.1	(33.3, 34.9)
Year								
2019	11.2	(8.1, 14.3)	11.0	(10.4, 11.5)	37.9	(33.3, 42.6)	34.3	(33.2, 35.4)
2021	21.0	(17.3, 24.8)	23.7	(22.7, 24.7)	38.8*	(34.0, 43.5)	34.0	(32.8, 35.2)
Gender								
Female	19.6	(15.7, 23.6)	18.4	(17.6, 19.1)	41.4	(36.8, 46.1)	34.1	(33.2, 35.0)
Male	13.4	(10.2, 16.6)	16.1	(15.4, 16.9)	35.6	(31.1, 40.1)	34.2	(33.1, 35.2)
Race/Ethnicity								
Non-Hispanic White	17.7	(14.8, 20.6)	19.7	(19.0, 20.4)	39.2	(35.0, 43.4)	36.9	(35.9, 37.8)
Non-Hispanic Black	15.5	(7.6, 23.4)	12.1	(10.5, 13.6)	38.9*	(28.2, 49.6)	27.3	(25.1, 29.5)
Non-Hispanic Other	14.5	(4.0, 25.0)	23.4	(21.2, 25.6)	41.8	(28.2, 55.5)	37.8	(35.3, 40.2)
Hispanic	11.6	(5.3, 17.9)	9.3	(8.3, 10.4)	32.4	(22.8, 42.1)	27.7	(26.1, 29.3)
Ages								
18 to 39	15.9	(11.8, 20.1)	15.3	(14.6, 16.0)	39.0*	(33.6, 44.4)	33.4	(32.4, 34.5)
40 to 50	17.8	(12.1, 23.4)	20.2	(19.0, 21.4)	37.2	(29.5, 45.0)	35.4	(34.1, 36.7)
51 to 64	16.1	(12.2, 20.0)	18.3	(17.2, 19.3)	38.3	(33.2, 43.4)	34.4	(33.1, 35.6)
Education								
Less than a high school	1.1	(−1.4, 3.6)	2.5	(1.7, 3.4)	29.3	(17.6, 41.0)	21.6	(19.3, 23.9)
High school diploma	7.5	(3.9, 11.2)	5.3	(4.8, 5.9)	32.5*	(26.8, 38.3)	24.7	(23.4, 25.9)
Some college	13.7	(9.9, 17.4)	11.7	(10.9, 12.4)	37.5*	(31.4, 43.6)	31.0	(29.9, 32.2)
Four-year degree	34.5	(29.3, 39.7)	30.8	(29.8, 31.8)	49.0	(42.3, 55.6)	44.1	(42.9, 45.3)

Sources: Current Population Survey (CPS) Disability Supplement, 2019 and 2021.

Chi-squared tests compare disability to no disability, * p<.05. There were no significant differences between disability and no disability groups for Formal WFH agreement.

NOTES: Includes the civilian non-institutional population of workers who were not self-employed, ages 18 to 64. Educational Attainment categories of “High school diploma” includes high school equivalent; “Four-year degree” includes Four-year degree and beyond. Titles were shortened to fit into the table

**Table 3:** Percentage of Workers who Work Remotely by Disability Status with Two Intersections *(American Community Survey, 2019 and 2021)*

	2019				2021			
	With Dis.	95% CI	No Dis.	95% CI	With Dis.	95% CI	No Dis.	95% CI
Disability	3.9	(3.7, 4.1)	4.0	(4.0, 4.1)	14.6	(14.3, 15.0)	17.4	(17.3, 17.5)
Intersections								
Female	4.8	(4.5, 5.1)	4.4	(4.3, 4.5)	16.6	(16.1, 17.1)	18.9	(18.7, 19.0)
Male	3.1	(2.9, 3.3)	3.6	(3.6, 3.7)	12.7	(12.3, 13.1)	16.1	(16.0, 16.2)
Non-Hispanic White	4.4	(4.2, 4.6)	4.7	(4.6, 4.7)	15.2	(14.8, 15.6)	18.6	(18.5, 18.8)
Non-Hispanic Black	2.8	(2.3, 3.3)	2.8	(2.7, 3.0)	14.7	(13.6, 15.8)	14.9	(14.6, 15.3)
Non-Hispanic Other	4.5	(3.8, 5.3)	4.1	(3.9, 4.2)	16.1	(15.1, 17.1)	24.2	(23.9, 24.5)
Hispanic	2.8	(2.4, 3.2)	2.6	(2.5, 2.7)	11.8	(11.0, 12.6)	11.1	(10.9, 11.3)
18 to 39	3.1	(2.8, 3.3)	3.3	(3.2, 3.3)	14.0	(13.5, 14.5)	16.6	(16.5, 16.7)
40 to 50	4.3	(3.9, 4.8)	4.8	(4.7, 4.9)	16.9	(16.1, 17.8)	19.2	(19.0, 19.4)
51 to 64	4.5	(4.3, 4.8)	4.8	(4.7, 4.9)	14.0	(13.5, 14.6)	17.4	(17.2, 17.5)
Less than a high school education	2.8	(2.3, 3.5)	2.2	(2.1, 2.4)	6.3	(5.6, 7.0)	5.4	(5.2, 5.6)
High school degree (or GED)	2.5	(2.2, 2.8)	2.4	(2.3, 2.4)	7.0	(6.6, 7.4)	7.3	(7.2, 7.5)
Some college	4.0	(3.7, 4.3)	3.5	(3.5, 3.6)	13.6	(13.0, 14.2)	12.7	(12.5, 12.8)
Four-year degree or beyond	6.1	(5.7, 6.5)	5.9	(5.8, 6.0)	27.5	(26.7, 28.4)	29.1	(28.9, 29.2)

Sources: American Community Survey (ACS) 2019 and 2021

NOTES: Includes the civilian non-institutional population of workers who were not self-employed, ages 18 to 64

**Table 4:** Percentage of Workers who Work Remotely by Disability Status with Three Intersections (American Community Survey, 2019 and 2021)

	2019				2021			
	With Dis.	95% CI	No Dis.	95% CI	With Dis.	95% CI	No Dis.	95% CI
Intersections								
Gender * Race								
Female *Non-Hispanic White	5.4	(5.1, 5.7)	5.1	(5.0, 5.2)	17.3	(16.7, 17.9)	19.7	(19.6, 19.9)
Female *Non-Hispanic Black	3.3	(2.6, 4.1)	3.3	(3.1, 3.5)	17.0	(15.7, 18.4)	17.6	(17.2, 18.0)
Female *Non-Hispanic Other	5.6	(4.5, 7.1)	4.4	(4.2, 4.6)	17.0	(15.6, 18.5)	24.2	(23.7, 24.6)
Female *Hispanic	3.6	(2.9, 4.3)	2.9	(2.7, 3.0)	13.6	(12.5, 14.9)	13.3	(13.1, 13.6)
Male *Non-Hispanic White	3.5	(3.2, 3.8)	4.3	(4.2, 4.4)	13.3	(12.7, 13.9)	17.6	(17.5, 17.8)
Male *Non-Hispanic Black	2.1	(1.6, 2.8)	2.3	(2.1, 2.5)	11.4	(9.7, 13.4)	11.8	(11.4, 12.2)
Male *Non-Hispanic Other	3.3	(2.5, 4.3)	3.8	(3.6, 4.0)	15.2	(13.8, 16.7)	24.2	(23.8, 24.6)
Male *Hispanic	2.1	(1.6, 2.6)	2.4	(2.2, 2.5)	9.9	(8.9, 11.1)	9.3	(9.0, 9.5)
Gender * Age								
Female *18 to 39	3.8	(3.5, 4.2)	3.6	(3.6, 3.7)	15.6	(15.0, 16.3)	18.1	(17.9, 18.3)
Female *40 to 50	5.3	(4.7, 6.1)	5.2	(5.1, 5.4)	18.7	(17.4, 19.9)	20.6	(20.3, 20.8)
Female *51 to 64	5.5	(5.0, 6.0)	5.1	(5.0, 5.2)	16.4	(15.8, 17.2)	18.8	(18.5, 19.0)
Male *18 to 39	2.4	(2.1, 2.7)	2.9	(2.8, 3.0)	12.3	(11.5, 13.0)	15.2	(15.0, 15.4)
Male *40 to 50	3.4	(2.9, 3.8)	4.3	(4.2, 4.5)	15.1	(14.2, 16.1)	18.0	(17.7, 18.2)
Male *51 to 64	3.7	(3.4, 4.1)	4.5	(4.3, 4.6)	11.8	(11.1, 12.5)	16.0	(15.8, 16.3)
Gender *Education								
Female *Less than a high school	3.9	(2.9, 5.2)	2.6	(2.4, 2.8)	7.9	(6.6, 9.5)	6.5	(6.1, 7.0)
Female *High school diploma	3.6	(3.1, 4.1)	2.9	(2.8, 3.0)	9.6	(8.8, 10.3)	9.8	(9.6, 10.1)
Female *Some college	4.8	(4.4, 5.3)	3.9	(3.8, 4.0)	15.2	(14.4, 16.0)	14.6	(14.4, 14.8)
Female *Four-year degree	6.5	(5.9, 7.1)	5.8	(5.7, 5.9)	27.2	(26.3, 28.2)	27.7	(27.4, 27.9)
Male *Less than a high school	2.1	(1.6, 2.7)	2.0	(1.8, 2.2)	5.1	(4.4, 5.9)	4.8	(4.5, 5.0)
Male *High school diploma	1.7	(1.5, 2.1)	1.9	(1.9, 2.0)	5.0	(4.5, 5.5)	5.5	(5.4, 5.7)
Male *Some college	3.2	(2.8, 3.6)	3.1	(3.0, 3.2)	11.8	(11.0, 12.7)	10.7	(10.5, 10.9)
Male *Four-year degree	5.7	(5.1, 6.4)	5.9	(5.8, 6.1)	27.9	(26.6, 29.2)	30.7	(30.4, 30.9)
Race*Age								
Non-Hispanic White *18 to 39	3.3	(3.0, 3.6)	3.7	(3.6, 3.8)	14.1	(13.5, 14.8)	17.6	(17.4, 17.7)
Non-Hispanic White *40 to 50	4.9	(4.4, 5.5)	5.6	(5.5, 5.8)	17.9	(16.9, 18.9)	20.7	(20.4, 20.9)
Non-Hispanic White *51 to 64	5.1	(4.7, 5.4)	5.5	(5.4, 5.6)	15.0	(14.2, 15.7)	18.7	(18.6, 18.9)
Non-Hispanic Black *18 to 39	2.3	(1.7, 3.0)	2.7	(2.5, 2.9)	13.8	(12.2, 15.6)	14.4	(14.0, 14.9)
Non-Hispanic Black *40 to 50	3.6	(2.5, 5.3)	3.2	(2.9, 3.4)	18.7	(16.0, 21.7)	16.2	(15.6, 16.8)
Non-Hispanic Black *51 to 64	2.7	(2.1, 3.6)	2.9	(2.6, 3.2)	12.9	(11.6, 14.4)	14.7	(14.2, 15.3)
Non-Hispanic Other *18 to 39	3.2	(2.3, 4.5)	3.6	(3.4, 3.8)	15.6	(14.1, 17.2)	24.4	(23.9, 24.8)
Non-Hispanic Other *40 to 50	4.6	(2.9, 7.3)	5.0	(4.7, 5.3)	17.8	(15.7, 20.0)	27.2	(26.6, 27.8)
Non-Hispanic Other *51 to 64	6.3	(4.7, 8.3)	4.3	(4.0, 4.6)	15.6	(13.9, 17.4)	20.1	(19.6, 20.6)
Hispanic *18 to 39	2.8	(2.2, 3.6)	2.3	(2.2, 2.4)	12.6	(11.4, 13.8)	11.0	(10.7, 11.2)
Hispanic *40 to 50	2.6	(2.0, 3.4)	3.0	(2.8, 3.2)	12.4	(10.9, 14.1)	11.6	(11.2, 12.0)
Hispanic *51 to 64	2.8	(2.2, 3.5)	2.9	(2.7, 3.1)	9.9	(8.7, 11.3)	10.8	(10.4, 11.3)
Race*Education								
Non-Hispanic White*Less than high school	2.7	(2.1, 3.5)	2.7	(2.5, 3.0)	6.8	(5.8, 8.0)	6.3	(6.0, 6.6)
Non-Hispanic White*High school diploma	2.7	(2.4, 3.2)	2.6	(2.5, 2.7)	6.7	(6.3, 7.2)	7.5	(7.3, 7.7)
Non-Hispanic White*Some college	4.4	(4.0, 4.7)	4.0	(3.9, 4.1)	13.4	(12.7, 14.1)	13.0	(12.8, 13.2)
Non-Hispanic White*Four-year degree	7.0	(6.4, 7.5)	6.5	(6.4, 6.6)	28.3	(27.3, 29.3)	28.8	(28.6, 29.0)
Non-Hispanic Black *Less than a high school	2.2	(1.1, 4.1)	1.9	(1.5, 2.3)	7.6	(5.4, 10.7)	6.6	(5.8, 7.6)
Non-Hispanic Black * High school diploma	1.9	(1.4, 2.6)	1.8	(1.6, 2.0)	7.8	(6.5, 9.5)	7.1	(6.7, 7.6)
Non-Hispanic Black *Some college	3.2	(2.4, 4.3)	2.7	(2.5, 2.9)	15.6	(13.9, 17.6)	13.0	(12.6, 13.5)
Non-Hispanic Black *Four-year degree	3.6	(2.7, 4.9)	4.3	(4.0, 4.7)	24.8	(22.3, 27.6)	26.1	(25.4, 26.7)
Non-Hispanic Other *Less than a high school	4.2	(2.4, 7.2)	2.5	(2.1, 3.0)	6.7	(4.8, 9.3)	7.1	(6.4, 8.0)
Non-Hispanic Other * High school diploma	3.4	(2.2, 5.2)	2.6	(2.3, 2.9)	6.3	(5.1, 7.7)	8.3	(7.9, 8.8)
Non-Hispanic Other *Some college	4.7	(3.5, 6.4)	3.5	(3.2, 3.8)	13.8	(12.3, 15.6)	13.9	(13.4, 14.4)
Non-Hispanic Other *Four-year degree	5.0	(3.8, 6.5)	5.0	(4.8, 5.2)	29.4	(27.0, 31.8)	35.7	(35.3, 36.1)
Hispanic *Less than a high school	3.0	(2.1, 4.2)	1.9	(1.8, 2.1)	5.2	(4.1, 6.5)	4.4	(4.1, 4.7)
Hispanic * High school diploma	1.8	(1.3, 2.5)	2.0	(1.8, 2.1)	7.5	(6.4, 8.7)	6.6	(6.3, 6.9)
Hispanic *Some college	2.9	(2.1, 3.8)	2.6	(2.4, 2.8)	12.7	(11.3, 14.2)	10.5	(10.1, 10.9)
Hispanic *Four-year degree	4.0	(2.9, 5.3)	4.2	(3.9, 4.5)	24.2	(21.7, 26.8)	23.8	(23.3, 24.3)
Age*Education								
18 to 39*Less than a high school degree	2.5	(1.7, 3.7)	2.2	(2.0, 2.5)	5.3	(4.2, 6.7)	5.3	(5.0, 5.6)
18 to 39* High school diploma	2.1	(1.8, 2.5)	2.1	(2.0, 2.2)	6.3	(5.7, 6.9)	6.7	(6.6, 6.9)
18 to 39*Some college	3.2	(2.8, 3.6)	2.9	(2.8, 3.0)	12.7	(11.8, 13.7)	10.8	(10.6, 11.0)
18 to 39*Four-year degree	4.6	(4.0, 5.2)	4.7	(4.6, 4.8)	28.0	(26.5, 29.6)	29.2	(29.0, 29.4)
40 to 50*Less than a high school	2.6	(1.7, 4.0)	2.1	(1.9, 2.3)	8.1	(6.6, 10.0)	5.1	(4.8, 5.5)
40 to 50* High school diploma	2.3	(1.8, 3.0)	2.5	(2.3, 2.6)	8.1	(6.9, 9.5)	7.7	(7.4, 8.0)
40 to 50*Some college	4.4	(3.7, 5.2)	4.2	(4.1, 4.4)	15.9	(14.7, 17.1)	14.6	(14.3, 15.0)
40 to 50*Four-year degree	7.1	(6.1, 8.3)	7.0	(6.8, 7.1)	28.5	(26.9, 30.2)	30.0	(29.7, 30.3)
51 to 64*Less than a high school	3.2	(2.6, 4.1)	2.3	(2.1, 2.6)	6.0	(5.0, 7.2)	5.8	(5.5, 6.2)
51 to 64* High school diploma	3.1	(2.6, 3.6)	2.8	(2.6, 2.9)	7.2	(6.5, 7.9)	8.1	(7.9, 8.3)
51 to 64* Some college	4.7	(4.2, 5.3)	4.4	(4.3, 4.6)	13.4	(12.5, 14.3)	14.8	(14.5, 15.0)
51 to 64*Four-year degree	6.9	(6.2, 7.6)	7.1	(6.9, 7.3)	26.3	(25.1, 27.6)	27.9	(27.5, 28.2)

Sources: American Community Survey (ACS) 2019 and 2021

NOTES: Includes the civilian non-institutional population of workers who were not self-employed, ages 18 to 64. Educational Attainment categories of “High school diploma” includes high school equivalent; “Four-year degree” includes Four-year degree and beyond. Titles were shortened to fit into the table

## References

[R1] ACS PUMS (2019). American Community Survey 2019 Data Dictionary. 1–146.

[R2] Ameri M, Schur L, Adya M, Bentley FS, McKay P, & Kruse D (2018). The disability employment puzzle: A field experiment on employer hiring behavior. ILR Review, 71(2), 329–364.

[R3] Bailey M, & Mobley IA (2019). Work in the intersections: A black feminist disability framework. Gender & Society, 33(1), 19–40.

[R4] Brault M. (2009). Review of changes to the measurement of disability in the 2008 American Community Survey. Census Working Papers. https://www.census.gov/library/working-papers/2009/demo/brault-01.html.

[R5] Brooks JD (2020). Workers with disabilities may remain unemployed long after the COVID-19 pandemic Issue Brief #30). Syracuse University, Lerner Center for Public Health Promotion and Population Health. https://www.maxwell.syr.edu/research/lerner-center/population-health-research-brief-series/article/workers-with-disabilities-may-remain-unemployed-long-after-the-covid-19-pandemic.

[R6] Brooks JD (2021). An intersectional analysis of Labor Market Outcomes. In Brown R, Maroto M, & Pettinicchio D (Eds.), The Oxford handbook of the sociology of disability. Oxford University Press. 10.1093/oxfordhb/9780190093167.013.32.

[R7] Brown RL, & Ciciurkaite G (2022). Precarious employment during the COVID-19 pandemic, disability-related discrimination, and mental health. Work and Occupations. 10.1177/07308884221129839.

[R8] Brown RL, & Moloney ME (2019). Intersectionality, work, and well-being: The effects of gender and disability. Gender & Society, 33(1), 94–122.

[R9] U.S. Bureau of Labor Statistics (2023). Persons with a disability: Labor force characteristics—2022. https://www.bls.gov/news.release/disabl.nr0.htm.

[R10] Coate P (2021). Remote work before, during, and after the pandemic (Quarterly Economics Briefing-Q4 2020). National Council on Compensation Insurance. https://www.ncci.com/SecureDocuments/QEB/QEB_Q4_2020_RemoteWork.html.

[R11] Cook L, von Schrader S, Malzer V, & Mimno J (2019). Unwelcoming workplaces: Bullying and harassment of employees with disabilities. In Bruyère SM (Ed.), Employment and disability: Issues, innovations, and Opportunities volumes (pp. 129–154). Labor and Employment Relations Associations Series.

[R12] Crenshaw K. (1989). Demarginalizing the intersection of race and sex: A black feminist critique of antidiscrimination doctrine, feminist theory, and antiracist politics. [1989]. Feminist legal theory (pp. 57–80). Routledge.

[R13] Crooks VA (2007). Women’s experiences of developing musculoskeletal diseases: Employment challenges and policy recommendations. Disability and Rehabilitation, 29(14), 1107–1116. 17612997 10.1080/09638280600948193

[R14] Davis DM (2021). Some Black women feel safer working from home and are opting out of office life to escape workplace racism. Insider. https://www.businessinsider.com/working-from-home-is-beneficial-to-some-black-women-2021-7.

[R15] Dorfman D. (2019). Fear of the disability con: Perceptions of fraud and special rights discourse. Law & Society Review, 53(4), 1051–1091.

[R16] U.S. Equal Employment Opportunity Commission (2003, February 3). Work at home/telework as a reasonable accommodation.https://www.eeoc.gov/laws/guidance/work-hometelework-reasonable-accommodation.

[R17] Erickson W, Lee C, & von Schrader S (2023). Disability statistics from the American Community Survey (ACS). Cornell University Yang-Tan Institute (YTI. http://www.disabilitystatistics.org.

[R18] Erkulwater JL (2018). How the nation’s largest minority became White: Race politics and the disability rights movement, 1970–1980. Journal of Policy History, 30(3), 367–399.

[R19] Fraser RT, Johnson K, Hebert J, Ajzen I, Copeland J, Brown P, & Chan F (2010). Understanding employers’ hiring intentions in relation to qualified workers with disabilities: Preliminary findings. Journal of Occupational Rehabilitation, 20(4), 420–426. 10.1007/s10926-009-9220-1. 19936892

[R20] Giovanis E, & Ozdamar O (2019). Accommodating employees with disabilities: The role of flexible employment schemes in Europe. https://papers.ssrn.com/sol3/papers.cfm?abstract_id=3441925.

[R21] Harlan SL, & Robert PM (1998). The social construction of disability in organizations: Why employers resist reasonable accommodation. Work and Occupations, 25(4), 397–435.

[R22] Hickox SA, & Liao C (2020). Remote work as an accommodation for employees with disabilities. Hofstra Lab & Emp LJ, 38, 25.

[R23] Igeltjørn A, & Habib L (2020). Homebased telework as a Tool for inclusion? A literature review of Telework, Disabilities and Work-Life Balance. In Antona M, & Stephanidis C (Eds.), Universal Access in Human-Computer Interaction. Applications and practice. HCII 2020 (12189 vol., pp. 420–436). Springer. Lecture Notes in Computer Science 10.1007/978-3-030-49108-6_30.

[R24] Inclusively (2022). The Immense Impact of Long COVID on Workers And What Employers Can Do About It. https://www.inclusively.com/immense-impact-of-long-covid-on-workers/.

[R25] International Labor Organization (ILO) (2023). Working Time and Work-Life Balance Around the World. Geneva: International Labour Office. https://www.ilo.org/global/publications/books/WCMS_864222/lang--en/index.htm.

[R26] Kanter AS (2022). Remote work and the future of disability accommodations. Cornell Law Review, 107. https://papersssrn.com/sol3/papers.cfm?abstract_id=4327135.

[R27] Kruse D, Park SR, van der Meulen Rodgers Y, & Schur L (2022). Disability and remote work during the pandemic with implications for cancer survivors. Journal of Cancer Survivorship, 16(1), 183–199. 35107797 10.1007/s11764-021-01146-z PMC8809229

[R28] Landes SD, Turk MA, McDonald KE, & Sabatello M (2020). Less worthy lives? We must prioritize people with intellectual and developmental disabilities in COVID-19 vaccine allocation (Issue Brief #42). Syracuse University, Lerner Center for Public Health Promotion and Population Health. https://www.maxwell.syr.edu/research/lerner-center/population-health-research-brief-series/article/less-worthy-lives-we-must-prioritize-people-with-intellectual-and-developmental-disabilities-in-covid-19-vaccine-allocation.

[R29] Lockhart PR (2019, August 16). How slavery became America’s first big business. Vox. https://www.vox.com/identities/2019/8/16/20806069/slavery-economy-capitalism-violence-cotton-edward-baptist.

[R30] Maroto M, & Pettinicchio D (2014). Disability, structural inequality, and work: The influence of occupational segregation on earnings for people with different disabilities. Research in Social Stratification and Mobility, 38, 76–92.

[R31] Maroto M, & Pettinicchio D (2015). Twenty-five years after the ADA: Situating disability in America’s system of stratification. Disability Studies Quarterly, 35(3), 1–43.

[R32] Maroto M, Pettinicchio D, & Patterson AC (2019). Hierarchies of categorical disadvantage: Economic insecurity at the intersection of disability, gender, and race. Gender & Society, 33(1), 64–93.

[R33] Mauldin L, Grossman B, Wong A, Miles A, Barnartt S, Brooks J, Frederick A, & Volion A (2020). Disability as an axis of inequality: A pandemic illustration. ASA Footnotes, 48(3), 20.

[R34] Orr AE, & Savage T (2021). Expanding access to and ensuring equity in the benefits of remote work following the COVID-19 pandemic. Journal of Science Policy & Governance, 18(4).

[R35] Ozimek A. (2020). The new geography of remote work. UpWork. https://www.upwork.com/research/new-geography-of-remote-work.

[R36] Paine N (2020, May 15). The industries hit hardest by the unemployment crisis. FiveThirtyEight. https://fivethirtyeight.com/features/the-industries-hit-hardest-by-the-unemployment-crisis/.

[R37] Pettinicchio D, Maroto M, & Brooks JD (2022). The sociology of disability-based economic inequality. Contemporary Sociology, 51(4), 249–270.

[R38] Robert PM, & Harlan SL (2006). Mechanisms of disability discrimination in large bureaucratic organizations: Ascriptive inequalities in the workplace. Sociological Quarterly, 47(4), 599–630. 10.1111/j.1533-8525.2006.00060.x.

[R39] Sabatello M, Landes SD, & McDonald KE (2020). People with disabilities in COVID-19: Fixing our priorities. The American Journal of Bioethics: AJOB, 20(7), 187–190. 10.1080/15265161.2020.1779396. 32716763 PMC7393634

[R40] Sanford C, Newman L, Wagner M, Cameto R, Knokey AM, & Shaver D (2011). The post-high school outcomes of young adults with disabilities up to 6 years after high school: Key findings from the National Longitudinal Transition Study-2 (NLTS2) (NCSER 2011–3004). National Center for Special Education Research. https://ies.ed.gov/ncser/pubs/20113004/pdf/20113004.pdf.

[R41] Schur LA, & Kruse D (2020). Coronavirus could revolutionize work opportunities for people with disabilities. The Conversation. https://theconversation.com/coronavirus-could-revolutionize-work-opportunities-for-people-with-disabilities-137462.

[R42] Schur LA, Ameri M, & Kruse D (2020). Telework after COVID: A silver lining for workers with disabilities? Journal of Occupational Rehabilitation, 30(4), 521–536. 33156435 10.1007/s10926-020-09936-5 PMC7645902

[R43] Shuey KM, & Jovic E (2013). Disability accommodation in Nonstandard and Precarious Employment arrangements. Work and Occupations, 40(2), 174–205. 10.1177/0730888413481030.

[R44] The Economic Daily (2017, October 17). Workers with a disability more concentrated in service occupations than those with no disability. U.S. Bureau of Labor Statistics, U.S. Department of Labor. https://www.bls.gov/opub/ted/2017/workers-with-a-disability-more-concentrated-in-service-occupations-than-those-with-no-disability.htm.

[R45] Travis MA (2021). A post-pandemic antidiscrimination approach to workplace flexibility. Wash U J L & Pol’y, 64, 203. https://openscholarship.wustl.edu/law_journal_law_policy/vol64/iss1/13/.

[R46] United States Census Bureau (2021). S1811 SELECTED ECONOMIC CHARACTERISTICS FOR THE CIVILIAN NONINSTITUTIONALIZED POPULATION BY DISABILITY STATUS. https://data.census.gov/table?q=United+States+disability&g=010XX00US&tid=ACSST1Y2021.S1811

[R47] Verbrugge LM, & Jette AM (1994). The disablement process. Social Science and Medicine, 38(1), 1–14. 10.1016/0277-9536(94)90294-1. 8146699

